# Antimicrobial Importance of Medicinal Plants in Nigeria

**DOI:** 10.1155/2020/7059323

**Published:** 2020-09-22

**Authors:** Harriet U. Ugboko, Obinna C. Nwinyi, Solomon U. Oranusi, Toluwase H. Fatoki, Conrad A. Omonhinmin

**Affiliations:** ^1^Microbiology Unit, Department of Biological Sciences, Covenant University, Ota PMB 1023, Nigeria; ^2^Biotechnology Cluster, Covenant University, Ota PMB 1023, Nigeria; ^3^Translational Bioinformatics Unit, Department of Biochemistry, Federal University, Oye Ekiti, Ekiti State, Nigeria; ^4^Biology Unit, Department of Biological Sciences, Covenant University, Ota PMB 1023, Nigeria

## Abstract

Despite the success of antibiotic discovery, infectious diseases remain the second leading source of death worldwide, while the resistance to antibiotics is among the significant problems in the twenty-first century. Medicinal plants are very rich in phytochemicals which can be structurally optimized and processed into new drugs. Nigeria enjoys a diverse collection of medicinal plants, and joint research has ascertained the efficacy of these plants. Plants such as guava (*Psidium guajava*), ginger (*Zingiber officinale*), neem (*Azadirachta indica*), and moringa (*Moringa oleifera*) have been found to exhibit broad range of antimicrobial activities. Studies on Nigerian plants have shown that they contain alkaloids, polyphenols, terpenes, glycosides, and others with possible therapeutic potentials. The antimicrobial activities of some new compounds such as alloeudesmenol, hanocokinoside, orosunol, and 8-demethylorosunol, identified from medicinal plants in Nigeria, are not yet explored. Further investigation and optimization of these compounds will facilitate the development of new sets of pharmacologically acceptable antimicrobial agents. This review study revealed the efficacy of medicinal plants as an alternative therapy in combating and curtailing the development and survival of multidrug-resistant pathogens coupled with the toxic effects of some antibiotics. Due to enormous therapeutic possibilities buried in medicinal plants, there is a need for more research into unique fingerprints and novel compounds that can provide cure to the neglected tropical diseases (NTDs) of humans and animals facing Africa, especially Nigeria.

## 1. Introduction

Infectious diseases have been consistently found to be among the leading causes of threat to global health. The World Health Organization (WHO) in 2013 reported that infectious diseases accounted for 61.7% (5.9 million) of the 9.6 million deaths in the sub-Saharan African region. Plants with medicinal value have found application in healthcare from the olden years. Globally, there are evidence-based studies to verify the efficacy of medicinal plants, and some of these shreds of evidence have provided insights into the synthesis of plant-based compounds with therapeutics application [1]. The annual global market value of medicinal plant products has exceeded $100 billion [2].

The “traditional or herbal medicines” are those originating from plant sources and are generally regarded as safe (GRAS) at the concoction dosage, based on their historical usage in various cultures [3]. Thus, plants remain the most abundant natural primary source of active drugs and are invaluable in the ethnomedical treatment of diverse ailments [4]. Medicinal plants are generally sources of various phytochemicals, some of which are usually responsible for their biological activities.

Traditional medicine as defined by the World Health Organization is the total of the knowledge, skills, and practices based on the theories, beliefs, and experiences indigenous to different cultures, whether explicable or not, used in the maintenance of health as well as in the prevention, diagnosis, improvement, or treatment of physical and mental illness. However, traditional, complementary medicine in Nigeria continues to thrive as it is commonly practised in other African nations as well as in Asia. Application of ethnomedicinal knowledge in the fields of biosciences for investigation of novel bioactive compounds as well as the polypharmacological formulation of plant extracts for use in primary healthcare has been the central interest in research [5].

Phytochemical screening of the medicinal plants is usually done against broad spectrum of microorganisms to ascertain their antimicrobial activities, based on the active constituents of the plants that are primarily secondary metabolites. The present occurrence of antimicrobial drug resistance by most bacteria has posed an enormous problem [6] and triggered the need for continuous research for better and safe therapeutic agents.

Most of the plants that found application in ethnomedicine have been documented based on their promising activities against multiple disease-causing microorganisms [7, 8]. Research efforts are expediting for better functional understanding of medicinal plants, and this has provided a model for about 25–50% of the marketed drugs [9]. Antimicrobial activities of useful plants vary; the majority act in synergy [10], reducing the side effect of synthetic drugs [11], while others act as quorum quenchers [12].

Moreover, this review summarized the salient information on the antimicrobial activity of medicinal plants in Nigeria and provided new bioactive components. This review explored research works on medicinal plants originating from Nigeria, and it highlights the constituents with potential therapeutics activities relevant to the treatment of microbial infections. The focus of this work is limited to the inhibitory effects of phytochemicals on diseases of microbial origin.

## 2. Phytochemical Constituents of Medicinal Plants

Medicinal plants contain bioactive organic chemical compounds often referred to as phytochemicals, which play a defensive role against major chronic diseases in both host-metabolic or genetic dysfunctional disease and infectious disease, and found in grains, vegetables, fruits, and other plant products [13–16]. Phytochemicals perform intermediary metabolic activities, and they function as primary metabolites such as fats and sugars found in all plants, while secondary metabolites are found in a smaller range of plants and provide specialized functions.

Secondary metabolites are biomolecules produced for sustainability or adaptation of plants in the environment as a result of many factors, such as protection from drought, pollination, and predation, but are not required for immediate survival of the plants [17]. Secondary metabolites and pigments, because of their healing effects in humans are processed into drugs such as inulin (dahlias plant), morphine and codeine (poppy plant), quinine (cinchona plant), and digoxin (foxglove plant) [18].

**Figure 1:**
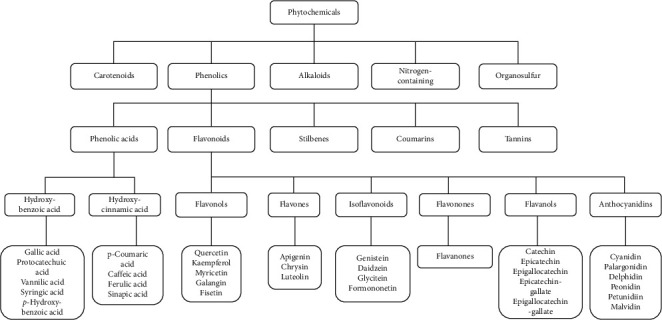
Classification of phytochemicals adapted from [15].

Screening of chemical composition of medicinal plants revealed that they contain different bioactive compounds which include saponins, tannins, and alkaloids [19]. The main functional classes of phytochemicals with therapeutic potential include antioxidants, anticancer agents, immunity-potentiating agents, detoxifying agents, and neuropharmacological agents [17]. As shown in Figure 1, each of the functional classes of the phytochemicals consists of a wide range of compounds at varying potency and sometimes with multiple functions [16]. Plants produce unique arrays of phytochemicals that frequently belong to existing biochemical motifs [20]. Among the bioactive compounds, triterpenoids provide anti-inflammatory activity and tannins possess astringent, anti-inflammatory, and antimicrobial activity properties [21]. Saponins often have medicinal features as blood cleansers, expectorants, and antibiotics. At the same time, alkaloids have significant effects on the central nervous system (CNS), and glycosides are known for their ability to increase the forces of systolic concentration [22].

### 2.1. Phytochemical Screening

The active ingredients of medicinal and aromatic plants extracts are in the roots, stems, bark, leaves, or flowers [17]. Most times, the actions of these active ingredients on the human CNS could be associated with ecological roles or biochemical identities in other plants and higher animals [23]. The significant steps to utilize these biologically active compounds from plant resources involve the extraction, primary screening, isolation, purification, and characterization as well as pharmacological and toxicological analyses [24].

Pharmaceutically, the extraction involves the separation of constituents of plant and microorganism tissues using selective buffer solution or solvents according to standard protocols [25, 26]. The crucial parameters that can influence the quality of an extract from the plant include parts used as a sample (leaf, bark, or root), solvent and its concentration used for extraction, and extraction method, while the effect of chemical constituents obtained from the extract depends on the nature of the plant material, its source, the extent of processing, and level of moisture, as well as particle size [27]. Factors that can influence the secondary metabolite composition and quantity in an extract include type and time of extraction, nature and concentration of the solvent, processing temperature, and polarity of analytes [27].

Different techniques, which include varieties of column chromatography, gas chromatography-mass spectrometry (GC-MS), Fourier-transform infrared spectroscopy (FTIR), ultraviolet (UV) spectrophotometry, and nuclear magnetic resonance (NMR), are often used stepwise for purification, identification, and structural characterization of various groups of phytochemical compounds in plants extracts [22, 24, 28].

### 2.2. Effect of Processing on Phytochemicals

The processing of medicinal plants can alter antioxidant activity by increase or decrease in efficacy. Methods of processing of medicinal plants can impact on the phytochemical constituent availability for pharmacological effect. Baba and Onanuga [29] found that different methods of processing sandpaper leaf (*Ficus exasperata*) extracts inhibited arginase and angiotensin-1-converting enzymes (ACE) activities in a dose-dependent manner. Moreover, their result showed that the soaking method produced significantly higher inhibition of both enzymes than other methods which include hand maceration, boiling, and blending. Previous studies show that the health benefits of the medicinal plant in mono- or poly-formulations are often a result of complex mixtures of phytochemicals in a holistic form which synergistically modulates multiple targets to produce overall therapeutic actions due to combined antioxidant properties [30–32]. The effects of processing methods (blanching, cooking or thermal processing, dehydration or drying, extrusion, and irradiation), other nonthermal processing (storage, carbon dioxide treatment), and other ingredients in the formulation on the phytochemicals have been extensively reviewed by [15]. However, during processing, naturally occurring antioxidants often undergo degradation such as Strecker degradation, as well as reactions such as Maillard reaction, leading to the formation of new compounds with lower or higher antioxidant activity, thereby accounting for the differences in the total phenolic contents and antioxidant activities in the processed when compared to unprocessed foods [33].

## 3. Medicinal Plants in Nigeria with Antimicrobial Activities

Despite the success of antibiotic discovery, infectious diseases are consistently ranked second among causes of death worldwide [34]. The pursuit of new compounds that have therapeutic potential for infectious diseases with no existing remedy such as Lassa fever and others focuses mainly on plants as the best reservoir of drug compounds (Table 1). The microbial resistance to antibiotics is among the significant problems in the twenty-first century and has necessitated the need for a continuous search for more potent and safe therapeutic agents [7].

### 3.1. Antibacterial Activity

Tuberculosis (TB) is a life-threatening disease caused by various *Mycobacterium* species and has been ranked among the leading causes of human death in the developing nations [51]. In 2013, WHO reported that approximately 8.6 million people incurred TB and 1.3 million died from it, with an estimated 450,000 new cases of multidrug-resistant TB worldwide. Chronic cough is a feature of many common respiratory diseases with the percentage of global occurrence in Oceania (18.1%), Europe (12.7%), America (11.0%), Asia (4.4%), and Africa (2.3%) [52]. Diarrhea is a killer disease; according to 2015 observatory data of WHO, the occurrence of childhood mortality in developing nations due to diarrhoeal disease was between 9% and 34%. Many enteric bacterial pathogens are responsible for diarrhea, which include bacteria such as *Vibrio cholera, Campylobacter jejuni, Helicobacter pylori, Salmonella typhi* or *paratyphi, Shigella flexneri*, *Clostridium difficile,* and Shiga toxin-producing *Escherichia coli.*

Findings of [53, 54] stated that the root and leaf extracts of *Terminalia glaucescens* show appreciable activity against *E*. *coli* and *S*. *typhi* [53]. The methanol extracts of three Nigerian medicinal plants have antimicrobial activity against five clinical bacterial isolates comprising two Gram-positive bacteria (*Bacillus subtilis* and *Staphylococcus aureus*) and three Gram-negative bacteria (*Pseudomonas aeruginosa*, *E*. *coli*, and *Klebsiella pneumonia*) organisms [7]. Acetone and ethanol extracts of bark of *Azadirachta indica* (A. Juss.), at a concentration of 25–400 mg/ml, showed significant antibacterial activity on all 14 strains of multidrug-resistant *Salmonella typhi* with zone diameter of 18–31 mm [55].

An investigation of the activity of aqueous and ethanolic extracts of *Zingiber officinale* and *Allium sativum* extracts on selected foodborne pathogens *(Salmonella species*, *Bacillus cereus*, *E*. *coli*, and *S*. *aureus*) showed multidrug resistance. *E*. *coli* was sensitive to aqueous extracts, but *S*. *aureus* and *Salmonella species* were sensitive to ethanol extracts [56]. The alcoholic extracts from the leaves of two *Diospyros* spp. (*D. barteri* and *D*. *monbuttensis*) showed potent antibacterial activity against a wide range of Gram-positive and Gram-negative bacteria, while two fungal species investigated in the study, *Aspergillus niger* and *Candida albicans*, were resistant to the extracts of both *Diospyros* spp. [57].

The result of *in vitro* study of water, methanol, chloroform, and petroleum ether extracts of *Senna alata* flowers, which examined antimicrobial properties at a final concentration of 500 *μ*g/mL, showed antimicrobial activities against clinical isolates of *S*. *aureus, C*. *albicans, E*. *coli, Proteus vulgaris, P*. *aeruginosa,* and *B*. *subtilis* [58]. *Heeria insignis* O. Ktze, a member of the family Anacardiaceae, is an indigenous African shrub used in the treatment of diarrhea, schistosomiasis, and venereal diseases. The methanol and dichloromethane extracts of the leaves of *H*. *insignis* possessed antibacterial and antidiarrheal activities, while methanol showed a more significant antibacterial activity than dichloromethane [59]. However, aqueous extracts of the stem bark of *Spondias mombin* have outstanding anthelmintic activity at low concentrations [3].

Preliminary investigation of the *in vitro* vibriocidal activities of three medicinal plants (traditional Ogi-tutu, *Psidium guajava,* and *Vernonia amygdalina*), using agar cup diffusion assay, showed that *V*. *amygdalina* has the highest ameliorative effects in the deterrence and cure of *V*. *cholerae* infection [60]. Moreover, a study has revealed the antibacterial property of *T*. *vulgaris* on multiple antibiotic-resistant *Vibrio fluvialis* and *Vibrio parahaemolyticus* isolated from shrimps using agar diffusion method [61]. The examination of *Argemone mexicana* L. for antimicrobial activity showed that the aerial and root extracts inhibited *B*. *subtilis* and *K*. *pneumoniae,* but *P*. *aeruginosa* and *S*. *aureus* were not prone to the aerial and root extracts.

In Nigerian ethnomedicine, *Annona senegalensis* Pers. (Annonaceae) has been used for the treatment of infectious diseases. Okokon et al. [62] investigated *A*. *senegalensis* using the GC-MS and agar-well-diffusion method and found that lipophilic fraction and kaurenoic acid from *A*. *senegalensis* root bark showed potent antibacterial activity. Furthermore, the results of *in vivo* and *in vitro* model investigations of the antidiarrheal properties of the stem bark extract of *A*. *senegalensis* using mice and isolated rabbit jejunum showed maximum inhibition at a dose of 10 mg/kg. At the same time, intestinal transit time decreased at concentrations of 0.2–3.2 mg/ml, and the extract lessened spontaneous contractions of the jejunum [63].

*Ficus exasperata* Vahl-Holl (Moraceae) leaves in West Africa are used for the treatment of infectious diseases and inflammatory conditions. However, a more thorough phytochemical analysis of the ethnomedicinal uses of *F*. *exasperata* has identified new compounds: apigenin C-8 glucoside, isoquercitrin-6-*O*-4-hydroxybenzoate, and quercetin-3-*O*-*β*- rhamnoside, as some of the constituents of this plant that inhibited the growth of Gram-positive organisms only [64].

### 3.2. Antimalarial Activity

Malaria, a significant threat to global health, is responsible for the death of millions of people predominantly in sub-Saharan Africa. The occurrence of multidrug-resistant malaria parasites has triggered efforts to develop combined formulations (such as artemether-lumefantrine and sulfadoxine-pyrimethamine) and continuous research toward discovering better therapeutics. An *in vitro* study by Oladele [65] on the sensitivity pattern of *Plasmodium falciparum* to *Diospyros monbuttensis* (“Egun eja”), *Momordica charantia* (“Ejirin”), and *Morinda lucida* (“Oruwo”) recorded the lowest antiplasmodial activity with the ethanolic extract of *M*. *lucida* (IC_50_ = 25 nM) while *D*. *monbuttensis* recorded the highest activity (IC_50_ = 3.2 nM).

A study on the anticoccidial effect of *M*. *lucida* on *Eimeria* parasites has suggested its potential as an anticoccidial drug for veterinary purposes, especially the poultry birds [66]. Furthermore, the triterpenes and phytosterols derived from *M*. *lucida* showed high binding affinity toward the selected histone deacetylases (class I HDAC and HDAC7 isoforms) and exhibited good druggable characteristics [67].

Oladele [65], investigated the antiplasmodial activity of crude n-hexane and ethanolic extracts of *M*. *oleifera* seeds using the cold extraction method and found the highest parasite inhibition activity in crude ethanolic extract. Besides, *Landolphia owariensis* P.Beauv., a member of the family Apocynaceae, is used in southeast Nigeria for the treatment of malaria. A study has shown that methanol fractions of *L*. *owariensis* leaf in early, established, and residual infections in *Plasmodium berghei*-infected albino mice have the most significant antiplasmodial activity in all the models carried out, due to alkaloids, flavonoids, saponins, and tannins present in the fractions [68].

*Allamanda cathartica* and *Bixa orellana* are antimalarial plants [69]. Moreover, *Alchornea laxiflora* (Benth.), a member of the family Euphorbiaceae, is used traditionally in the treatment of malaria in Nigeria. Furthermore, the study has shown that root extract *A*. *laxiflora* exerted significant antimalarial activity against *P*. *berghei* infection while ethyl acetate fraction exerted the highest activity against chloroquine-sensitive (Pf3D7) and -resistant (PfINDO) strains of *P*. *falciparum* infection in mice [70]. *Enantia chlorantha* has significant utilization in traditional medicine for the treatment of several diseases which include malaria. However, another study has suggested that oral administration of *E*. *chlorantha* at relatively high doses may produce severe toxic effects [71]. A study on the acute and subacute toxicity of the medicinal plant *E*. *chlorantha* carried out in mice showed a mean lethal dose (LD_50_) of 0.7 g·kg^−1^ for ethanolic but 43.65 g·kg^−1^ for aqueous preparations [72].

*Cajanus cajan* (L.) is a member of the family Fabaceae and possesses antimalarial properties. The result of the *in vitro* investigation of crude methanolic extract of *C*. *cajan* leaves, using the multiresistant strain of *P*. *falciparum* (K1) and combination of chromatographic techniques, identified cajachalcone (2′,6′-dihydroxy-4-methoxy chalcone) from the ethyl acetate fraction, as one of the biologically active constituents [73].

*Azadirachta indica* (A. Juss.) belongs to the family Meliaceae, which is a common plant especially in Africa, and extracts from parts of this plant have shown pharmacological activities which include antiplasmodial activity [74]. However, the antimalarial activity of *A*. *indica* is yet to be fully ascertained, this calls for in-depth research on antimalarial activity of *A*. *indica*. However, an *in silico* study [75] has identified margolonone, nimbinone, and nimbione as the highest functional compounds that can modulate the activity of *P*. *falciparum* heat shock protein 90 (PfHsp90).

Research has shown that *Allophylus africanus* extracts curbed parasitemia caused by intraperitoneal administration of erythrocytes of *P*. *berghei* (NK-65) in mice, and it was found that flavonoids, saponins, tannins, and carbohydrates are predominant in all parts of *A*. *africanus* [76]. Moreover, four new compounds, hanocokinoside, allotaraxerolide, alloeudesmenol, and alloaminoacetaldehyde, have been identified as some of the chemical constituents of *A*. *africanus* [42]. Three lignans, (+) isolariciresinol, helioxanthin, and justicinol, have been isolated from *Justicia flava* VAHL leaves [77]; in addition, 8-demethylorosunol and orosunol were reported as two new 1-aryl-2, 3-naphthalide lignans from *J*. *flava* root [78]. Therefore, further investigation and optimization of these new compounds can help in the design and production of a new set of efficacious and safe antimicrobial agents.

### 3.3. Antifungal Activity

Infections caused by fungi are termed fungal infections or mycoses. Fungal infections have been considered a serious health problem and life-threatening diseases in recent years, especially in immunodeficiency conditions [8, 79]. Severe fungal diseases result from other health challenges such as human immunodeficiency virus (HIV), asthma, cancer, organ transplantation, and corticosteroid treatments [80]. In Nigeria, fungal infections (cryptococcal antigenemia, subclinical histoplasmosis) have been implicated in HIV/AIDS patients and neonatal intensive care babies [81, 82]. In Cameroun, esophageal candidiasis, cryptococcal meningitis, *Pneumocystis* pneumonia, disseminated histoplasmosis, and invasive aspergillosis were prevalent in adults, while tinea capitis was prevalent among school children ([83]. In Mozambique, disseminated *Emergomyces* and recurrent *Candida* vulvovaginitis were common among HIV patients [84]. Some hospitalized patients are at risk of contracting fungal infections [85]. Besides, emerging and reemerging fungal infections due to recent therapies for autoimmune and cancer-related diseases (hematopoietic stem cell transplant) are becoming public health concern [86, 87]. Different parts of *Calotropis procera*, a flowering plant that belongs to the family Asclepiadaceous, have been utilized in traditional medicine for the treatment of infections which include eczema, cutaneous infections, leprosy, and syphilis, as well as malaria. In a study on antifungal activity of *C*. *procera*, there was complete inhibition of *Microsporum* and *Trichophyton* species after ten days of inoculation with water extract at different concentrations [88].

Crude methanolic extract of *Spondias mombin* (bark and leaves) was found to exhibit anticandidal effects with diameters of 11.00 ± 0.47 mm and 15.00 ± 0.47 mm, respectively. The extracts have varying degrees of phytochemical compositions such as terpenoids, alkaloids, glycosides, saponin, and flavonoids [89]. *Psidium guajava* extracts were active on *Candida albicans* isolates from caries infected patients [90]. Additionally, *Alchornea laxiflora* leaf extracts have antibacterial and antifungal activities due to flavonoids, alkaloids, saponins, tannins, and reducing sugars as major phytochemicals [36].

### 3.4. Antiviral Activity

Human immunodeficiency virus type 1 (HIV-1) encodes reverse transcriptase which functions in the process of the viral genome reverse transcription, a fundamental step in the HIV-1 replication cycle and promising target in the antiretroviral drug development [91]. A study conducted in Nigeria [47] on the antiviral activities of 27 medicinal plant extracts, belonging to 26 different plant species, against echovirus 7, 13, and 19 serotypes (E7, E13, and E19, respectively) revealed the highest antiviral activity from methanolic extract of *Macaranga barteri* leaves on E7 and E9, respectively, followed by *Ageratum conyzoides* leaves extract on E7 and E19 and *Mondia whitei* leaves extract on E7 and E19. In China, *Rheum palmatum* and *Rheum officinale* extracts along with their main single isolated constituents' anthraquinone derivatives inhibited both HIV-1 reverse transcriptase-associated DNA polymerase (RDDP) and ribonuclease H activities [92]. The screening of some plants showed antiretroviral activities. For instance, *Ancistrocladus korupensis* [93] and *Ancistrocladus* congolensis [94] produced michellamine A and B [95], *Ancistrocladus congolensis* [94].

## 4. Mechanisms of Action of Medicinal Plants

The rare occurrence of infectious diseases in wild plants serves as a fundamental indication of the presence of competent defence mechanisms. Different mechanisms of action through which phytochemicals can exert antimicrobial activities include (i) inhibition of the activity of enzymes and toxins, (ii) damage of the bacterial membrane, (iii) suppression of virulence factors, (iv) formation of biofilm, (v) inhibition of protein synthesis, and (vi) quorum quenching [96]. The mode of action of tannins is based majorly on their ability to bind proteins, thereby inhibiting cell protein synthesis [97–99]. The intervention of quorum sensing is in three levels: (i) signal synthesis, (ii) signal sequestration, and (iii) signal reception [100].

### 4.1. Synergistic Activity of Antimicrobial Medicinal Plants and Antibiotics

Antibiotics have been effective in treating infectious diseases, but resistance to these drugs has led to the emergence of new and the reemergence of old infectious diseases. The development of resistance to antibiotics in bacteria has been found possible due to three reasons: (i) direct destruction or modification of the antibiotic by enzymes produced by the organism, (ii) modification of target resulting in a reduction in the efficiency of binding of the drug; and (iii) efflux of antibiotic from the cell [101–103].

One of the strategies used to overcome resistance mechanisms in combination therapy includes combined active drug or polyherbal formulations [10]. The use of natural products in combination with antibiotics to enhance treatment efficacy is a new strategy developed to overcome the problem of antibiotics resistance [104]. The presence of many bioactive compounds in a medicinal plant provides a synergistic therapeutic effect, but when used simultaneously with standard drugs, they often improve the activity of the drug and will confront problems of toxicity and overdose [13].

A combination of *Helichrysum pedunculatum* leaf extracts with antibiotics used against wound infection associated bacteria resulted in about 60% synergistic effect. Hemaiswarya et al. [105] investigated the antiplasmodial activities of various combinations of *Murraya koenigii* leaf, *Artocarpus altilis* stem bark [2], *Nauclea latifolia* root, and *Enantia chlorantha* stem bark, with standard drugs. They observed a significant reduction in the protective, chemosuppressive, and curative actions of *N*. *latifolia* coadministered with standard drugs. Besides, *N*. *latifolia* or *M*. *koenigii* in combination with other plants produced a synergistic effect, unlike using each plant alone. Furthermore, *E*. *chlorantha* with *A*. *altilis* or *N*. *latifolia* enhanced their respective prophylactic or curative activities and served as promising combinations for the treatment of malaria [2]. Coadministration of *Carica papaya* and *Vernonia amygdalina* plants in ameliorating *Plasmodium* infection in mice showed synergistic effects with significant percentage suppression of parasite load within three days of clinical therapy [106].

## 5. Future of Medicinal Plants

The future of medicinal plants is promising, considering the vast number of medicinal plants that are yet to be selected and investigated for their phytochemical compositions. Medicinal plants have provided an avenue for understanding the scaffold for synthetic drug design and development. In addition, the future of medicinal plants will influence the medical practice, considering the occurrence of new pathogens and diseases, which calls for alternative or complementary medicine [11]. The rising tide in antibiotic resistance by microorganism has raised signiﬁcant concern in the medical field and, thus, an urgent demand for the discovery of safe, natural compounds in this postgenomic era. The use of medicinal plants as nutraceuticals and functional foods is on the increase, as means of ensuring preventive medicine and finding a solution to this global concern of evolution of drug-resistant microorganisms [1].

## 6. Conclusion

This review report has revealed the efficacy of medicinal plants as an alternative therapy in tackling the development and spread of multidrug-resistant pathogens coupled with the toxic effects of some antibiotics. However, this study shows the lack of adequate characterization of the chemical composition of the antimicrobial medicinal plants in Nigeria. Only a few of the medicinal plants studied in Nigeria have their bioactive components investigated using advanced analyses such as GC-MS and HPLC. The absence of such analyses makes the claims from ethnomedicinal and phytochemical screening studies invalidated. Due to enormous therapeutic possibilities buried in the medicinal plants, there is a need for research into unique fingerprints and novel compounds that can provide cure to the neglected tropical diseases (NTDs) endemic in Africa and especially in Nigeria. Moreover, there is a need for innovative investigation of novel natural bioactive compounds for the development of chemical libraries useful for drug discovery and development pipelines. A better understanding of the underlying mechanism of the synergy of two or more combinations of phytochemicals at a molecular level will decrease the use of antibiotics and help to prevent and overcome drug-resistant pathogens.

## Figures and Tables

**Table 1:** Summary of selected antimicrobial medicinal plants in Nigeria.

SN	Botanical name of the medicinal plant	Major therapeutic application	References
1	*Argemone mexicana, Ficus exasperate, Persia Americana, Alchornea laxiflora, Crinum jagus, Adansonia digitate, Citrullus colocynthis, Cola nitida, Dorstenia prorepens, Echinacea purpurea, Spondias mombin, Annona senegalensis, Vernonia amygdalina, Thymus vulgaris*	Antibacterial	[35–40]

2	*Enantia chlorantha, Azadirachta indica, Justicia flava, Landolphia owariensis, Cassytha filiformis, Morinda lucida, Allamanda cathartica, Allophylus africanus, Clerodendrum capitatum, Bixa Orellana, Senna alata*	Antibacterial antiparasitic antifungal	[35, 37, 40–43]

3	*Mangifera indica, Myristica fragrans, Cajanus cajan Gossypium arboretum, Amaranthus spinosus, Heeria insignis, Momordica charantia, Diospyros monbuttensis, Morinda lucida, Calotropis procera*	Antiparasitic	[35, 37, 44, 45]

4	*Ageratum conyzoides, Macaranga barteri, Mondia whitei*	Antiviral	[46–50]
